# Exploring how triads of people living with dementia, carers and health care professionals function in dementia health care: A systematic qualitative review and thematic synthesis

**DOI:** 10.1177/1471301220915068

**Published:** 2020-03-25

**Authors:** Remco Tuijt, Jessica Rees, Rachael Frost, Jane Wilcock, Jill Manthorpe, Greta Rait, Kate Walters

**Affiliations:** University College London, UK; King’s College London, UK; University College London, UK

**Keywords:** dementia, health care, triad, qualitative review, thematic synthesis

## Abstract

**Background:**

Many qualitative studies report the post-diagnostic care experiences of carers and people living with dementia; however, this is not often accompanied by opportunities to hear the corresponding views of their health care professionals and how this triadic relationship functions. The aim of this review was to identify and thematically synthesize the experiences of health care services reported by people living with dementia, their carers and health care professionals.

**Methods:**

Medline, PsycINFO, Embase and CINAHL were searched from inception to 31 July 2019 for qualitative research including people living with dementia, carers and health care professionals. Data were coded and thematically synthesised using NVivo.

**Results:**

Of 10,045 search results, 29 papers relating to 27 studies were included in the final synthesis, including 261 people living with dementia, 444 carers and 530 health care professionals. Six themes emerged related to the functioning of a dementia care triad: (1) involving the person living with dementia, (2) establishing expectations of care and the roles of the members of the triad, (3) building trust, (4) effective communication, (5) continuity of care and (6) understanding the unique relationship dynamics within each triad.

**Discussion:**

The interactions and complexity of triadic dementia care relationships further our understanding of how to improve dementia care. Awareness of possible diverging attitudes highlights areas of necessary improvement and further research into facilitating engagement, such as when multiple professionals are involved or where there are mismatched expectations of the roles of triad members. In order to operate efficiently as a triad member, professionals should be aware of how pre-existing relations can influence the composition of a triad, encourage the involvement of the person living with dementia, clarify the expectations of all parties, establish trusting relationships and enable communication within the direct triad and beyond.

## Introduction

The care and treatment of people living with dementia aims to improve or sustain quality of life (Zabalegui et al., 2014), with pharmacological treatment or psychosocial interventions targeting cognitive or behavioural symptoms (Rabins et al., 2017). Best practice recommendations for pharmacological treatment exist (Fillit et al., 2006), but there is a major focus on non-pharmacological care due to limited suitability and efficacy of medication (Gitlin et al., 2012). Guidelines on dementia care encourage tailoring care to individuals’ needs and wishes (Fazio et al., 2018; National Institute for Health and Care Excellence (NICE), 2018).

Living as well as possible with dementia may be facilitated by the support of health and care professionals and a family member or someone close to the person living with dementia. In this paper, the term ‘carer’ refers to family members or friends who support a person living with dementia. The person living with dementia, the carer and health care professional may all contribute to the establishment and efficacy of support for the person living with dementia, and can be referred to as members of a triad (Fortinsky, 2001). As providers of health care for a person living with dementia can differ greatly, including but not limited to general practitioners (GPs), medical specialists or nurses (Jensen & Inker, 2015), this range is acknowledged in this review which focuses on the provision of health care and does not confine this to one provider.

Kitwood’s (1993) theoretical model of care advocated treating an individual with dementia as an active recipient of care and encouraged those providing care to reframe their perception of well-being for a person living with dementia. The ethos of person-centred care was developed to encompass four specific elements: valuing people living with dementia and those who care for them, treating people as individuals, looking at the perspective of the person living with dementia and encouraging a positive social environment (Brooker, 2003). Despite the widespread uptake of these principles, the implementation of person-centred care varies in practice (Olsson et al., 2013). Evolution of person-centred care also includes ‘relationship centred care’, where the interactions between individuals serve as the foundation of care (Adams & Gardiner, 2005; Nolan et al., 2004).

Critiques of person-centred care have highlighted a lack of proper definitions and a focus on independence and autonomy that may overlook the intricacies and importance of relationships to an individual (Ryan & Nolan, 2019), but proponents have argued that family members and significant others are included within person-centredness (Edvardsson et al., 2010). Still, the link between personhood and the relationships of the person living with dementia is under-researched, despite calls for person-centred care to be integrated with a broader approach to dementia care that addresses relationship dynamics (Smebye & Kirkevold, 2013). More recently developed frameworks of dementia care have included a focus on both person-centredness and relationships (Lord et al., 2019), but a further development of the understanding of the complexities of relationships within a dementia care triad is needed, particularly outside the context of long-term residential care.

In practice, there is often ‘fragmented care’ (Robinson et al., 2010), and the metaphor of a dementia care ‘journey’ is often used (Teel & Carson, 2003). Much research has analysed specific parts of this ‘journey’, including the diagnosis and disclosure of dementia and the effect of this on care provision and receipt (Bamford et al., 2004; Bunn et al., 2012) with some of this informed by people living with dementia. Prorok et al. (2013)'s meta-ethnography of 46 qualitative studies of dementia care urged professionals to be continuously person-centred, but they did not compare patient, carer and health care professional perspectives. As the knowledge base of care for people living with dementia continues to grow, so too does the need to research under-explored areas, such as comparative analysis of the experiences of individuals and their carers and professionals. Previous research concerning the health care triad has generally excluded individuals living with dementia as they were deemed unable to fully engage (Laidsaar-Powell et al., 2013).

However, bringing together the experiences and perspectives of the person living with dementia, their carer and health care professional may prompt greater understanding of the dynamic nature of their relationships and how these may facilitate or hinder optimal dementia care. This review aims to identify and synthesize the qualitative research involving the dementia health care triad where it concerns post-diagnostic treatment or health care of people living at home with dementia. We use the term health care in a broad sense to identify experiences related to dementia that may take place in different care environments (e.g. social care), but which do relate to the health care of the individual. We have excluded young onset dementia as the needs and experiences of those diagnosed before the age of 65 years or similar have been acknowledged to be greatly different from older age groups (Greenwood & Smith, 2016).

## Methods

We conducted a systematic review of qualitative studies following Cochrane guidelines for qualitative reviews (Higgins, 2011), with a thematic synthesis. The review protocol is registered on PROSPERO (CRD42019135584).

### Search strategy

We originally searched MEDLINE, PsycINFO, Embase and CINAHL, from inception to 31 January 2019. Searches were not restricted to any language or date of publication. Target searches combined terms and Medical Subject Headings related to (1) dementia with (2) qualitative research terminology and (3) health care experiences using Boolean operators. The full search strategy is available in online Appendixes A and B. Relevant papers and other reviews were reference checked for both forward and backward citations and grey literature searches using Google Scholar were used to identify any further unidentified papers (Haddaway et al., 2015). Inclusion and exclusion criteria are summarised in Table 1. In line with methodological expectations for systematic reviews (Chandler et al., 2013), an update search was conducted with the original search terms on all databases up to 31 July 2019.

**Table 1.** Inclusion and exclusion criteria.

Inclusion criteria	Exclusion criteria
Main topic of people living with dementia in the community	Mild cognitive impairment or other condition as main focus, dementia with intellectual disability, young onset dementia, long-term residential care, hospital or inpatient experiences
Qualitative research	Quantitative, survey, closed questionnaire, reviews, protocols, editorials, commentary
Concerning treatment/management of dementia	Concerning diagnosis, disclosure, research participation, scale development
Focused on the health care of the person living with dementia after diagnosis	Carer focused, practitioner focused, no health care listed as main outcome
Includes people living with dementia, carers and health care professionals	Not including all members of the triad

### Study screening and data extraction

One author (RT) screened all titles and abstracts of the identified studies in accordance with the inclusion criteria, and a second reviewer (JR) conducted a 10% independent inter-rater reliability check. No discrepancies were found. All full texts were assessed for inclusion by two authors (RT, JR) for all members of the health care triad and a focus on an element of post-diagnostic care. Data were extracted (RT) from the final studies included Author, Year, Country, Topic, Participants, Relation between participants established (Network), Data collection method (Collection) and Data analysis method (Analysis).

### Methodological quality

The qualitative version of the Critical Appraisal Skill Programme (CASP, 2018) was used to assess methodological quality of the included studies by two authors (RT, JR) independently, with any discrepancies resolved through discussion. Quality ratings informed data synthesis and were not used as exclusion criteria.

### Synthesis

Included full-text studies were imported into NVivo 11 for thematic synthesis (NVivo qualitative data analysis software, 2015). Views of each member of the triad were established separately where reporting was sufficient to determine distinctions. Data from members outside of the health care triad (e.g. healthy older adults) were not included in the analysis. The primary qualitative data included within the published papers, the themes reported and supporting text as well as their conclusions were coded (RT) where they covered health care experiences. The coded data were analysed for themes related to the functioning of the health care triad, which were inductively derived and grouped in a hierarchical structure in order to achieve thematic synthesis (Thomas & Harden, 2008). Further synthesis of themes was achieved through discussion with the wider multi-disciplinary research team.

## Findings

### Search results

**Figure 1.**
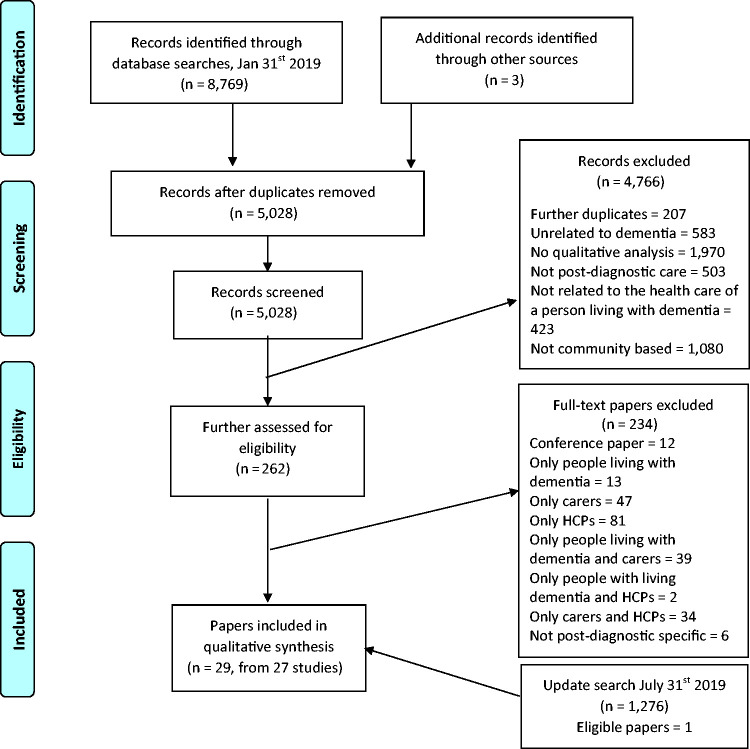
PRISMA flow diagram (Moher et al., 2009) of included studies. HCP: health care professional.

A total of 8769 records were identified from the original database searches. After removing duplicates, initial screening of abstracts and titles, 262 full texts were further assessed for inclusion of all members of a triad. At this stage, 234 full texts were excluded, leaving 28 papers covering 26 studies for inclusion (see Figure 1). The update search carried out six months later identified one additional study that was included in this first review, leading to a final total of 29 included papers concerning 27 studies.

### Characteristics of included studies

Out of 27 studies, 15 collected data through interviews only, four used focus groups only, while eight used different methods of data collection for different members of the triad. Most (12) studies took a clear ‘network approach’, which involved identifying other members of the same triad with the person living with dementia as the common link. The most frequently used method of data analysis was thematic analysis, followed by content analysis. In total, the included participants across all studies were 261 people living with dementia (median 7 per study), 444 carers (median 11.5) and 530 health care professionals (median 12). The health professionals interviewed included GPs, pharmacists, nurses, care workers, consultants and case managers. Participants who were not a direct member of a triad (*n* = 52) included healthy older adults, policymakers and charity representatives (data not included in analysis). Table 2 contains a full list of the characteristics of the studies.

**Table 2.** Characteristics of included studies.

Author and country	Topic	Participants	Triad recruitment	Collection (composition when using mixed methodology)	Analysis
1. Andersen et al. (2008) Canada	Medication expectations	Four people living with dementia, four lay caregivers,three geriatric consultant physicians, four community clinic nurses, four pharmacists, four policymakers	Y	Interviews	Data reduction, display, interpretation and verification method
2. Bogardus et al. (1998) USA	Decision making	Eight people living with dementia, 12 primary family caregivers, six physicians, three case managers	Y	Interviews	Constant comparative method
3. Bowes & Wilkinson (2003) UK	South Asian care experiences	One person living with dementia, four families and carers, two GPs, two CPNs, two consultants, two NHS ethnic minority mental health projects, three specialised voluntary service providers	Y	Interviews	Thematic analysis
4. Bronner et al. (2016) Germany	Decision making	Five people living with dementia six relatives, three physicians, six social education workers, two professional legal guardians, one nurse specialising in palliative care, one private carer	N	Interviews	Content analysis
5. Bunn et al. (2015) UK	Contextualisation of results of a systematic review	Three people living with dementia, 12 carers, six dementia service providers, five older people	Undefined	Focus groups, interviews (three carers and one person living with dementia)	Framework coding
6. Clarke et al. (2010) UK	Risk	Four people living with dementia, four family carers, one OT, one community practitioner, one CPN, one day care staff member	Y	Repeated interviews	Symbolic interactionism
7. Di Gregorio et al. (2015) Canada	Rural experiences	Two people living with dementia, 15 care partners, 37 health and social service providers, 17 community members	Undefined	Individual and group interviews	Constant comparative method
8. Dickins et al. (2018) Australia	Risk	Seven people living with dementia, 22 carers, eight registered nurses, 23 staff (senior nurses, social workers, community nursing management team) 20 healthy older people	Undefined	Interviews (people living with dementia, carers, nurses and staff), focus group (carers)	Thematic analysis
9. Foley et al. (2017) Ireland	Primary care educational needs	Five people living with dementia, 12 family carers, 14 GPs	N	Interviews	Thematic analysis
10. Forbes et al. (2011) Canada	Rural experiences	Three people living with dementia, three spouse caregivers, nine adult children, two grandchildren, three home care providers	Y	Interviews	Thematic analysis
11. Forbes et al. (2013) Canada	First Nation information sharing	Two people living with dementia, three care partners, two HCPs	Y	Repeated interviews	Grounded theory
12. Gilmour et al. (2003) UK	Risk	10 People living with dementia, 12 family members, nine care staff, six GPs	Y	Interviews	Template approach
13. Groen van de Ven et al. (2017) Netherlands	Decision making	23 People living with dementia, 44 informal caregivers, 44 professionals	Y	Interviews	Content analysis
14. Groen van de Ven et al. (2018) Netherlands	Decision making	23 People living with dementia, 44 informal caregivers, 44 professionals (same as Groen van de Ven 2017)	Y	Interviews	Content analysis
15. Low et al. (2013) Australia	Community care	One person living with dementia, 31 carers, 32 service providers, four policy representatives	Undefined	Interviews	Thematic analysis
16. Maidment et al. (2017) UK	Medication management	Four people living with dementia, 11 informal carers, 16 health/social care professionals	Undefined	Interviews	Framework analysis
17. Martin et al. (2013) UK	Self-management	Seven people living with dementia two family members, two charity representatives (AS), eight health care professionals	Undefined	Individual and group interviews	Thematic content analysis
18. Newton et al. (2016) UK	Assistive technology	13 People living with dementia, 18 current carers, eight former carers, 11 GPs, six GP trainees	Undefined	Interviews	Thematic analysis
19. Poppe et al. (2013) UK	Advance care planning	12 People living with dementia, eight carers, six staff	Y	Interviews	Constant comparison method
20. Post et al. (2001) USA	Medication	Three people living with dementia, four caregivers, 20 professionals	Undefined	Focus groups	Thematic analysis
21. Quinn et al. (2013) UK	Care relationships	Six people living with dementia, six caregivers, three admiral nurses	Y	Interviews	Interpretive phenomenological analysis
22. Risco et al. (2015) Spain	Care experience	Seven people living with dementia, 11 family caregivers, four PCPs, four geriatricians, two neurologists, three PC nurses, two hospital nurses, two PC social workers, two hospital social workers	N	Focus groups	Content analysis
23. Rothera et al. (2008) UK	Evaluation of specialist home care	27 People living with dementia, 18 family carers, 17 home care workers, 20 health/social care workers	Undefined	Interviews (*all*), small group interviews (*people living with dementia*), focus groups (*carers and home care workers*)	Soft systems methodology
24. Spector et al. (2011) UK	Care experiences	17 People living with dementia, 14 carers, seven facilitating staff	Y	Focus groups and interviews (*members of all groups took part in both*)	Framework analysis
25. St-Amant et al. (2012) Canada	Decision making	Nine people living with dementia, 25 family caregivers, 10 formal health care providers, seven community case managers	Y	Repeated interviews	Critical ethnography, Lofland data analysis
26. Stephan et al.(2018) Germany, Ireland, Italy, Netherlands, Norway, Portugal, Sweden, UK	Care experience	51 people living with dementia, 96 informal carers, 114 health care professionals	Undefined	Focus groups	Content analysis
27. Tilburgs et al. (2018) Netherlands	Advance care planning	Nine people living with dementia 13 family carers 10 GPs three case managers, two practice nurses/case managers (dual function)	Undefined	Interviews, focus group (*case managers and practice nurses only*)	Content analysis
28. Toot et al. (2013) UK	Crisis management	18 People living with dementia 15 family carers, 19 health care professionals (OTs, Admiral nurses, day hospital managers, HTT managers, psychologists and psychiatric nurses)	Undefined	Focus groups	Thematic analysis
29. Ward-Griffin et al. (2012) Canada	Care experience	Nine people living with dementia, 25 family caregivers, 10 formal health care providers, seven case managers *(Same as* St-Amant et al., 2012)	Y	Repeated interviews, focus group (*case managers only*)	Critical ethnography, Lofland data analysis

AS: Alzheimer’s Society; CPN: community psychiatric nurse; GP: general practitioner; HCP: health care professional; HTT: home treatment team; NHS: National Health Service; OT: occupational therapist; PC: primary care; PCP: primary care physician.

Note: Terms regarding carers and health care professionals are derived from the papers as provided.

### Methodological quality

CASP ratings ranged from 7 to 10 out of 10, indicating general moderate to high levels of quality in reporting qualitative research. The median of the ratings was 8, with most studies not reporting on the relationship between the researcher and the participant. Three studies received a 10 rating (Bunn et al., 2015; Gilmour et al., 2003; Quinn et al., 2013). The full ratings can be found in online Appendix C.

### Synthesis

The studies included reported on several different aspects of care and included a range of health care professionals. This section presents the themes that emerged, with the supporting primary data reported by papers in italic quotes, and the authors’ (of the papers) interpretations as plain quotes. The six themes regarding the functioning of a dementia triad comprise: (1) active participation and autonomy, (2) expectations of care and of the role in the triad, (3) building relationships and trust, (4) communication, (5) continuity of care and (6) relationship dynamics.

#### Active participation and autonomy

Most papers explored the ways people living with dementia were said to be able, or allowed, to take an active part in the triad communications and decision-making. The perceived capability of the person living with dementia by other members of the triad, as well as their perceived insight into their condition, was a main contributory factor to their participation in these activities. When the health care professional perceived the person living with dementia to be lacking insight less sensitive topics were discussed, there was a propensity to target the carer in consultations, there could be exclusion of the person living with dementia and restrictions could be applied to the person living with dementia (Dickins et al., 2018; Forbes et al., 2011; Maidment et al., 2017; Martin et al., 2013; Poppe et al., 2013; Tilburgs et al., 2018). As an example:

… it was often assumed – particularly by nurses – that those who have a diagnosis by deﬁnition had lost their capacity to make decisions about not only their health but other aspects of their life. (Dickins et al., 2018, p. 77)

However, when the person living with dementia was involved, it led to an increased feeling of being in control for the person living with dementia, less miscommunication and maintained their involvement in their care (Forbes et al., 2011; Groen van de Ven et al., 2018; St-Amant et al., 2012; Stephan et al., 2018).

People living with dementia expressed a desire to remain independent, in control and involved, which at times, according to others, could lead to denial of how severe their symptoms were, or refusing care because it was seen as a threat. To improve independence, activities were modified in order to keep them accessible, and ‘acceptable’ risks were tolerated if taking them was thought to improve mental and physical wellbeing (Dickins et al., 2018; Forbes et al., 2013; Groen van de Ven et al., 2017; Martin et al., 2013; Stephan et al., 2018). While most papers included notions of respecting autonomy, some people living with dementia reported feeling overlooked:

*I was present during the consultation, but I wasn’t able to participate actively* – Person living with dementia. (Bronner et al., 2016, p. 6)

The justification for excluding a person living with dementia by the other members of the triad related to the perception of the person living with dementia’s deteriorating cognitive ability:

*I ﬁnd it difﬁcult to take the initiative. Because you take things away from him, you know? You are going to decide and do this and that. You don’t want to do more than just mediate. But it becomes more and more you taking the lead about what he can and cannot do, kind of an executive role* – Carer. (Groen van de Ven et al., 2017, p. 1332)

There were also instances where a carer taking over for the person living with dementia was contested by another member of the triad as it seemed to detract from the capabilities of the person living with dementia:

*She’s taken things oﬀ him that he’s quite capable of doing or could do in a fashion, even if it’s not perfect. It’s her letting him do it and it not be perfect that’s probably the crux of it – Admiral (community dementia) Nurse*. (Quinn et al., 2013, p. 595)

Overall, encouraging people living with dementia to be involved was thought to be beneficial by all members of the triad, especially in the early stages of the disease when planning ahead. Despite the possibility of being distressing or dispiriting, involvement reassures, supports and helps other members of the triad, especially carers, feel justified in the decisions that are made then and in the future (Bronner et al., 2016; Forbes et al., 2013; Poppe et al., 2013).

#### Expectations of care and of the role in the triad

A disrupter in the functioning of the triad was mismatched expectations, of either the care needed for the person living with dementia or the role that each member of the triad should and would want to take. One of the more commonly mentioned areas where care expectations varied was medication (Andersen et al., 2008; Post et al., 2001). Specifically, the expectations of the benefits of medication did not always align between people living with dementia and other members of the triad:

The consensus among professionals was that many of the expressions of optimism [by the people with dementia and carers] were unrealistic. They urged caution for both persons with AD [Alzheimer’s disease] and their family caregivers, who may wrongly think they have a miracle cure and are, therefore, headed for a ‘hard fall.’ (Post et al., 2001, p. 83)

Personalisation of care was expected in line with notions of person-centred care and mentioned by all members of the triad. Personalised care had a positive impact on the person living with dementia and facilitated relationship building between all members of the triad (Bowes & Wilkinson, 2003; Di Gregorio et al., 2015; Forbes et al., 2013; Groen van de Ven et al., 2018; Low et al., 2013; Maidment et al., 2017; Martin et al., 2013; Risco et al., 2015; Stephan et al., 2018; Tilburgs et al., 2018). Where there was no personalisation, care was found inadequate and substandard (Ward-Griffin et al., 2012).

A strong building factor was enabling socialisation of the person living with dementia, as it combated loneliness, helped individuals find support, maintain a sense of identify and give them a sense of achievement (Bunn et al., 2015; Clarke et al., 2010; Forbes et al., 2011; Low et al., 2013; Spector et al., 2011; Stephan et al., 2018). People living with dementia reported feeling supported by contact with other people living with dementia:

*So yeah I think it helped all of us to know that we’re on the same boat on the same road, yes that was a very good part of it.* – Person living with dementia. (Spector et al., 2011)

Many studies reported that carers and people living with dementia felt there was a lack of information regarding available dementia services, the progression and symptoms of dementia and the legal and financial issues that may arise. Lack of information led to delayed or less service use, which increased the chance of risk and reduced coping strategies (Bunn et al., 2015; Di Gregorio et al., 2015; Foley et al., 2017; Low et al., 2013; Newton et al., 2016; Poppe et al., 2013; Quinn et al., 2013; Risco et al., 2015; Stephan et al., 2018; Ward-Griffin et al., 2012). Health care professionals highlighted that the heterogeneity of dementia makes it difficult to give exact information, which led to little confidence among some professionals to discuss such matters with people living with dementia for fear of them developing unrealistic expectations (Foley et al., 2017; Martin et al., 2013; Poppe et al., 2013; Stephan et al., 2018). Equally important was the way professionals presented possible developments:

*By giving expectations [of care] that are not real you get the patient and family against you immediately.* – Health care professional. (Risco et al., 2015, p. 227)

The right time to initiate domestic, personal or replacement care was often thought to be earlier than people living with dementia or carers might wish, as it encouraged planning, and enabled professionals to monitor and adjust decisions (Groen van de Ven et al., 2017; Poppe et al., 2013; Stephan et al., 2018; Tilburgs et al., 2018). Finding the right element of timing was also relevant to professionals feeling they needed to match the information provided to the stage of the disease, as presenting information could be stressful if done too early, but enabled further in-depth topics to be broached if done as and when the other members of the triad felt comfortable (Groen van de Ven et al., 2018; Poppe et al., 2013; Risco et al., 2015; Tilburgs et al., 2018). The timing as well as the content of information as such was important for the health care professional to consider:

*So it’s ﬁnding that medium, that middle ground where everything’s honest and accurate, but it’s not going to really hurt them, and it’s not going to make them believe things are other than what they really are*. – Health care professional. (Forbes et al., 2013, p. 368)

Regarding the role of each member in the triad, an initial barrier reported by carers and health care professionals at times was the person living with dementia and their acceptance of the diagnosis or need for care:

*… he never accepted that he was the one that needed the help –* Carer. (Bunn et al., 2015, p. 745)

When the person living with dementia did not accept their diagnosis, it disrupted the workings of professionals and the initiation and establishment of a triad, to a greater extent when the carer also did not recognise a need for formal care (Bowes & Wilkinson, 2003; Poppe et al., 2013; Stephan et al., 2018).

The role the carer was expected to take was discussed by carers themselves and by professionals. Often professionals acknowledged the view that carers were essential members of the triad:

*One of the big learnings I’ve had is the carer support and how important carer support is in the management of the patient* – GP. (Foley et al., 2017, p. 6)

The perceptions of the carer role could be further developed when some professionals viewed carers:

… as recipients of services in their own right as well as key partners in delivering care. (Low et al., 2013, p. 93)

Nonetheless, first and foremost, professionals felt it necessary to understand and address the carer’s expectations of the person living with dementia if these were unduly influential:

*She [carer] had unrealistic expectations at times of how he [person living with dementia] might understand and how he might communicate his own thoughts and feelings. I felt that some of her responses simply weren’t helping* – Admiral Nurse. (Quinn et al., 2013, p. 597)

Carers themselves sometimes felt like they had failed if paid care was taken on and some saw professionals as interfering (Bowes & Wilkinson, 2003; Dickins et al., 2018; Gilmour et al., 2003; Maidment et al., 2017; Stephan et al., 2018). From the perspective of the carers, taking over some of their relatives’ decisions was seen as part of their ‘duty’:

*Sometimes I feel fed up but what can I do? That is my duty … I forget my medicine but I never forget his* – Carer. (Maidment et al., 2017, p. 931)

Professionals were aware of carers feeling a sense of duty and at times counted upon it as part of an asset-based approach that emphasized unpaid care and viewed paid care as ‘supplementary’:

*I have a new [patient] and the ﬁrst thing that I did is to try to rally every family member … and gather as much family or friends that you can to start oﬀ with and focus on and get as much care in there as needed* – Case manager. (Ward-Griffin et al., 2012, p. 4)

The role of health care professionals was complex, as for example, some GPs felt that their support for the person living with dementia and their family could take on a counselling role (Foley et al., 2017), but there were also comments that disagreed with the idea that there was a therapeutic expectation of the health care professional role (Martin et al., 2013). People living with dementia and carers appreciated professionals who were able to work in the home environment as it provided context (Maidment et al., 2017; Risco et al., 2015).

The professionals most represented were GPs, but some papers did not specify beyond the term ‘health care professional’. It was acknowledged that people living with dementia often engaged with multiple professionals:

*We all seem to do a little bit of dementia each, but we don’t have perfectly dedicated people and if we do they’re very secondary care positioned*. – GP. (Newton et al., 2016,  p. 5)

But while having multiple professionals involved could complicate expectations and responsibilities of the professionals (Gilmour et al., 2003; Risco et al., 2015), one study observed that:

… different participants [professionals] often held different goals for care of the same patient. To the extent that these different goals simply reflect the different skills that people bring to a clinical setting, these differences may enhance care. (Bogardus et al., 1998, p. 679)

#### Building relationships and trust

Health professionals explicitly talked about the need to build trust with other members of the triad, which was related to more successful negotiation and planning of long-term goals, a shared sense of responsibility and improved relationships in the triad. In addition, it enabled all members to feel more comfortable sharing their views, which improved information sharing (Forbes et al., 2011, 2013; Gilmour et al., 2003; Groen van de Ven et al., 2017; Rothera et al., 2008; Tilburgs et al., 2018), as articulated by a carer:

*He (person living with dementia) did not have to be afraid anymore. He did not have to worry. He did not have to be nervous If he couldn’t remember something, well … he could get his thoughts of his mind so to speak … . There was a trusting relationship which was beautiful to see* – Carer. (Tilburgs et al., 2018, p. 4)

In some cases, there were reports of distrust by carers or people living with dementia of the professionals:

*My sister and I went to talk with [the case manager]. My mother absolutely did not trust this lady.* – Carer. (Groen van de Ven et al., 2018, p. 853)

Distrust, in some cases, was understood as a fear of becoming too reliant on professionals:

As a note of caution, some participants spoke of how support services can ‘ …  foster dependency’ (Martin et al., 2013, p. 487).

In building relationships, the most variable factor discussed by professionals and carers was who takes on the role of the carer and how. Identifying one carer could simplify interactions by having a single contact point for the professional, but the previous history of the relationship of that carer and person living with dementia needed to be taken into consideration, as well as the wider family network that may want to be involved (Bronner et al., 2016; Groen van de Ven et al., 2017; Quinn et al., 2013; St-Amant et al., 2012; Ward-Griffin et al., 2012). The involvement of a carer often positively benefited the person living with dementia, but could lead to carer stress.

#### Communication

All papers described on-going communication as facilitating care provision and acceptability. However, often the initial communication was considered by carers to be insufficient, which led to confusion around dementia symptoms and progression (Bowes & Wilkinson, 2003; Maidment et al., 2017; Newton et al., 2016; Risco et al., 2015). Professionals recognised the need for establishing good communication with other members of the triad, but also for improving communication between all members as:

… there is not only insufficient communication between persons with AD [Alzheimer’s Disease] and their relatives, but also between persons with AD and GPs. (Bronner et al., 2016, p. 7)

When the professional navigated communication, they could find common ground for care decisions to be made, as making them was challenging when previous communication strategies between members of the triad were not optimal (Groen van de Ven et al., 2017, 2018). This could be done by engaging the other members of the triad so:

… the participants feel that the process of deliberating potentially conﬂicting perspectives and interests is shared when they have a sense of working together in making decisions. (Groen van de Ven et al., 2018, p. 853)

Communication also extended to situations beyond the direct triad and, as noted above, might involve, for example, inter-professional communications and carers communicating with other family members:

*I don’t want it to be just me. I think it should be like a committee of the family. Like I don’t want them to all say, ‘Alright, what are you going to do?’ I said, ‘No, we all have to make this [decision] as a family.’* – Carer. (Forbes et al., 2013, p. 370)

Professionals communicating well with each other improved the support and treatment for the person living with dementia and seemed to facilitate appropriate and timely resource allocation (Maidment et al., 2017; Risco et al., 2015; Stephan et al., 2018). When communication was perceived by people living with dementia to be negative, it could limit the effective workings of a triad:

*I said to my own GP, I actually don’t want to see these doctors anymore because they are patronising*. – Person living with dementia. (Stephan et al., 2018, p. 9)

Where the person living with dementia had difficulty communicating effectively, due to, for example, their symptoms, this led to fewer contributions by the person living with dementia and increased carer stress. Changing communication strategies between the professional and the person living with dementia to shorter questions or taking more time, for example, could improve involvement, and in some cases assist carers in changing their communication towards the person living with dementia (Bowes & Wilkinson, 2003; Quinn et al., 2013; Tilburgs et al., 2018).

#### Continuity of care

All members of the triad regarded continuity (of both staff and care) as an important relationship factor. Continuity, for example, of a key contact, was perceived by all members of the triad to help establish successful and stable relationships and facilitate continuous access to care (Bunn et al., 2015; Forbes et al., 2013; Groen van de Ven et al., 2018; Low et al., 2013; Newton et al., 2016; Rothera et al., 2008; Stephan et al., 2018; Toot et al., 2013). A professional working with First Nation populations in rural Canada mentioned:

*You need continuity of care; that is number one. They have to get to know the person that they are working with, because there’s been so much distress in the past. Once they form that relationship with that person or those persons then it goes great. But if you have a continual change over and over and over and over, they don’t want to know. They get fed up. They get turned off. They take off. They won’t be there … that’s the key. You have to form a relationship* – Health care professional. (Forbes et al., 2013, p. 336)

The continuity provided by having contact with the same professional was expressed as a benefit by people living with dementia:

… continuity of staff was essential as familiarity reduced the potential for increased confusion for people with dementia, and allowed for a stable service relationship. (Low et al., 2013, p. 94)

It was also described as affecting the effectiveness of interventions:

Carers stressed the importance of having continuity when dealing with health and social care staff because it can very often impact on whether or not the crisis is effectively resolved. (Toot et al., 2013, p. 334)

Carers and people living with dementia reported that if there were too many professionals offering advice and getting involved, it could lead to confusion over tasks and professional involvement (Gilmour et al., 2003; Risco et al., 2015). Continuity was reduced when there was a lack of services, which resulted in increased risk of harm, and carers finding themselves stretched in order to provide more care (Stephan et al., 2018; Toot et al., 2013).

#### Relationship dynamics

The ways in which members of the triad worked together, all three in agreement or not, received limited attention, as few papers took a defined comparative triadic viewpoint. However, it was possible to discern some instances where one member of the triad, or two together, held specific viewpoints that opposed the other or stood out as individual. The interviews with health care professionals had the sharpest focus on presenting information and managing the triad:

Professionals can operate strategically once they are aware of the interactions within the care network, and can thus navigate between the network members to ﬁnd common grounds. For instance, they can function as a bridge between care network members who have difﬁculties in discussing their situation together. (Groen van de Ven et al., 2017, p. 1332)

People living with dementia seemed to be more focused on their own capabilities and independence, positively and negatively:

Equally, the care-recipients [people living with dementia] will balance these views against their own perceptions of their abilities and may not want to follow the suggestions made. (Quinn et al., 2013, p. 599)

Carers expressed concerns about accessing and organising help, sometimes leading to them ignoring their own health:

… many family members reported feeling unheard when they expressed concerns or an inability to continue providing care. (Ward-Griffin et al., 2012, p. 4)

It seemed common for people living with dementia and their carers to agree with each other; at times against the advice of the professional member of the triad. The initiation of services, for example, was questioned, with professionals recommending earlier initiation of services but:

… people with AD (Alzheimer’s Disease) saw it as their relatives’ responsibility to care for them and also the relatives felt committed to care for their family members. (Bronner et al., 2016, p. 8)

Fewer mentions were made of circumstances where the professional and the person living with dementia concurred and the carer did not, but an example was when the professional thought they needed to advocate the perspective of the person living with dementia. For example, a staff member reported trying to initiate a conversation about advance care planning:

*I think the client would have been quite open to the discussion but the daughter was quite … that wasn’t somewhere that she wanted to do and she was, so we didn’t*. – Professional. (Poppe et al., 2013, p. 4)

Carers and professionals collaborated more often, generally in situations where they thought that the person living with dementia lacked insight, for example:

… the informal caregivers and professional report that people with dementia may overestimate their capabilities. (Groen van de Ven et al., 2018, p. 851)

The relationship dynamics that were available in the studies reviewed were limited in reports of triadic perspectives. However, they provide some context for the previously described themes where, for example, there were more mentions concerning just one member of the triad rather than another.

## Discussion

To the best of our knowledge, this review is the first to systematically search the literature contrasting the views and experiences of the triad of people living with dementia, their carers and health care professionals. To better understand how to support people living with dementia, it may be helpful to investigate how such triads operate, as well as what supports or what disrupts their relationships. In summary, encouraging the autonomy and involvement of the person living with dementia was thought important to keep them engaged with the other members of the triad. Expectations related to care were essential to clarify, specifically concerning personalising or creating individual support in a timely fashion. This was related to the relationships and trust between the members of the triad, but often directed towards the health care professional, who could take on navigating and streamlining communication. Continuity of care supported the triadic relationships that had formed, also through continuity of staff. Finally, the relationship dynamics enhanced coalitions between members of the triad in opposing another member or helped establish context. In these ways, the relationships between the different members of the triad were established, navigated and strengthened. The themes that had the widest range of coverage included the autonomy and participation of the person living with dementia, and the expectations and acceptance of care. Fewer studies explored relationship dynamics between members of the triad or established how and why the building of relationships and trust was important.

For people living with dementia, this review reaffirms that they should be active participants in their care in line with policy and professional guidance (Fazio et al., 2018; NICE, 2018). Health and care decisions can and should be made with their involvement, and this may need encouragement and support in advance care planning and follow up after a diagnosis. There is increasing evidence that many people living with dementia can make informed decisions (Horton-Deutsch et al., 2007), which supports the principle of people living with dementia being an active member of their triad. However, some accounts of people living with dementia not being involved were reported, where the person living with dementia felt they were either not included as a member of the triad or they felt excluded by the other two members. Professionals could pay attention to dynamics that lessen the involvement of the person living with dementia, and approach the person living with dementia as a potentially active and able individual who is disabled by their impairment, other conditions, and environment.

For carers, this review’s findings underlines their contribution to providing information about care for people living with dementia, and initiate and negotiate services for the person living with dementia while also providing their own care. Other research has shown the benefits of engaging and involving carers in interventions, and encourages their support in order to improve quality of life for both the person living with dementia and themselves (Robinson et al., 2010). This review focussed on care provision for the person living with dementia and did not cover carer support or interventions. The role of the carer in the triad however seems pivotal, as their relationship with both the person living with dementia and the professional facilitates engagement. Carers influenced the working of the triad by having the most contact and engagement with the other two members. As with all triads, this meant that coalitions could form against other members, similar to the understanding of relationship-centred care (Adams & Gardiner, 2005). For people living with dementia without a carer, special attention should be made to encourage and establish good relationships with professionals, especially key workers or case managers.

As for health care professionals, their initial engagement with the members of the triad was usually with a pre-existing dyad, as they would be brought into an existing relationship of a carer and a person living with dementia. They thus needed to define their role, which should cover their own relationships with other professionals that might engage more often with the other members of the triad. The nature of care systems means some professionals spend more time with the person living with dementia and the carer than others, and where they are able to work with them in their home, this seemed to facilitate the working of the triad. Case managers and Admiral Nurses (specialist community mental health nurses), for example, showed better engagement than GPs who reported not enough time with patients, or non-specialist homecare workers who may not be able to provide continuity or much time.

Overall, care for a person living with dementia and the areas in which the triad operates will evolve as the dementia progresses. Some professionals will be able to provide effective care and address different topics, while many people living with dementia and their carers will find themselves in multiple triads, something which was not well represented in the included studies. The suggestion that a central point of contact, or care coordinator, can improve care is understandable if multiple triad professionals in a triad are exchanged for a singular one. However, the multiple inter-relationships that exist within wide-ranging health and care support services will still be important and influential even if a single care coordinator is introduced, complicating any model of care that does not consider more than three possible members of a ‘triad’. As continuity is an important relationship factor, for some people living with dementia, the establishment of a triad that builds on existing relationships may be effective. It is important to remember that some people living with dementia will not have carers and for them a dyadic relationship with their key health or care professional may be more relevant. Where health care professionals can navigate and establish an ongoing triad, they should encourage the involvement of the person living with dementia, understand the expectations of all involved parties, establish trusting relationships and communicate within the direct triad and beyond.

Finally, the complexity of the dementia care triad shown in the themes identified as part of this review provides context when applying or developing relevant models of care, whether this takes a person- or relationship-centred care approach. Frameworks of both types of care have had difficulties in their effective implementation (Venturato et al., 2013), and so the understanding of how relationships within a dementia care triad are developed and managed in different care contexts may assist development of supportive health care for a person living with dementia.

### Strengths and limitations

The strength of the findings of this review relies on the rigorous approach taken to the identified studies, the rich range of data identified and the depth of the analysis. No conclusions can be drawn regarding areas of care or involvement of professionals where there was no previous qualitative research involving triads, such as where other health conditions were involved or intersected with the lived experiences of dementia. The triad literature was limited concerning pharmacists and homecare workers, and areas of practice such as advance care planning and management of non-cognitive symptoms. As this study had a focus on the triadic relationship in dementia care, there was no limit to the range of health care professionals that were included. Some studies were clearer about who was consulted, and while the findings appear in general to be transferable across the various professions, there was limited data and clarity of reporting in included studies to draw specific conclusions about individual types of health care professionals. In addition, people living with dementia who do not have an identifiable carer, are, de facto, not represented in this study, although one study (Gilmour et al., 2003) focused on those living alone with dementia and included family members who lived elsewhere. As such, future research should capture the experience of people living with dementia who live without a carer.

In order to identify the widest range of eligible studies, broad search terms were used to identify any qualitative research on experiences of health and care for people living with dementia. Only after screening was a specific triadic nature applied as a criterion in order to include research using different terminology or a unique approach. Two studies were identified that had split findings into two papers, but these were excluded as they did not present findings with a comparative or synthesising approach.

### Implications for practice and research

Guidelines that encourage every effort to involve the person living with dementia in care provision seem implicitly to suggest a triad approach. The influence of expectations and understanding on the relationship between the person living with dementia and the carer affects the work of health care professionals and *vice versa*. In order to recognise a person living with dementia as an active or influential participant in their care, professionals need to understand the relationship between the carer and the person living with dementia, and the impact of intervening on this dyad and on themselves. The effort it takes to establish an effective triad may be assisted by the continuity and consistency of a health care professional, especially where various professionals are involved and communication becomes more complex and more necessary. The relationship factors central to engaging a person living with dementia and a carer should be taken into consideration by all professionals, emphasizing the difficulty in achieving good care due to the uniqueness of each triad. Future research should chart the workings and interplay of the relationship dynamics between all members of the triad over time in order to provide more understanding of best practice in dementia care, including where people living with dementia may be in multiple triads with several carers or several health care professionals.

## Conclusion

The qualitative literature concerning the experiences of people living with dementia, carers and professionals provides an understanding of the intricacies of establishing a working care relationship between the members of a dementia care triad. This includes where members of the triad are agreed as well as when they differed, for example, when multiple professionals or carers are involved or when expectations are mismatched. The health care professional that takes on the role of navigating a triad in order to provide care can strengthen the dementia care triad by involving the person living with dementia, ensuring continuity of care and establishing expectations of care, effective communication and trust. Professionals who engage with triads should be aware of the unique composition of each triad and the relationship dynamics that affect this. Future research should be encouraged to take a triadic view and look to explain differences in perspectives in order to improve the workings of the dementia care triad.

## Supplemental Material

DEM915068 Supplemental Material - Supplemental material for Exploring how triads of people living with dementia, carers and health care professionals function in dementia health care: A systematic qualitative review and thematic synthesis Supplemental material, DEM915068 Supplemental Material for Exploring how triads of people living with dementia, carers and health care professionals function in dementia health care: A systematic qualitative review and thematic synthesis by Remco Tuijt, Jessica Rees, Rachael Frost, Jane Wilcock, Jill Manthorpe, Greta Rait and Kate Walters in Dementia
