# Supplementary material for: Exploring how triads of people living with dementia, carers and health care professionals function in dementia health care: A systematic qualitative review and thematic synthesis
Source: Dementia (London). 2020 Mar 25;20(3):1080–104. doi: 10.1177/1471301220915068 (PMC8047709; doi:10.1177/1471301220915068)
Supplement: DEM915068 Supplemental Material - Supplemental material for Exploring how triads of people living with dementia, carers and health care professionals function in dementia health care: A systematic qualitative review and thematic synthesis [file DEM915068_Supplemental_Material.pdf]

Appendix

A. Search terms table

Search target	MESH	Free text
1. Dementia	Dementia<br>Frontotemporal Dementia<br>Dementia, Multi Infarct<br>Dementia, Vascular<br>Alzheimer Disease<br>Lewy Body Disease	dement*<br>alzheimer*<br>((memory adj2 problem*) or (memory adj2 difficult*) or (memory adj2 issue*))
2. Qualitative research	Qualitative Research<br>Evaluation Studies<br>Focus Groups<br>Interview	(view* or perspective* or opinion* or attitude or qualitative or ethnograph* or evaluat*)<br>(focus group* or interview*)
3. Health care experiences	Attitude to Health<br>Professional-Patient Relations<br>Professional-Family Relations<br>Attitude of Health Personnel<br>Patient-Centered Care	(improv* or experience* or attitude* or percept* or perspect* or perciev* or view* or value* or belief*) adj3:<br>Healthcare*<br>Care<br>Treatment*<br>Service*<br>Management*

B. Database Searches

Medline:

1. Dementia/
2. Frontotemporal Dementia/
3. Dementia, Multi Infarct/
4. Dementia, Vascular/
5. Alzheimer Disease/
6. Lewy Body Disease/
7. dement*.ti.
8. alzheimer*.ti.
9. ((memory adj2 problem*) or (memory adj2 difficult*) or (memory adj2 issue*)).ti.
10. or/1-9
11. Qualitative Research/
12. Evaluation Studies/
13. Focus Groups/
14. Interview/
15. ((focus adj2 group*) or interview*).ti,ab.
16. (view* or perspective* or opinion* or attitude* or qualitative or ethnograph*).ti,ab.
17. or/11-16
18. exp Attitude to Health/
19. Professional-Family Relations/
20. exp Professional-Patient Relations/
21. Attitude of Health Personnel/
22. exp Patient-Centered Care/
23. ((improv* or experience* or attitude* or percept* or perspect* or perciev* or view* or value* or belief*) adj3 care).ti,ab.
24. ((improv* or experience* or attitude* or percept* or perspect* or perciev* or view* or value* or belief*) adj3 service*).ti,ab.
25. ((improv* or experience* or attitude* or percept* or perspect* or perciev* or view* or value* or belief*) adj3 practice*).ti,ab.
26. ((improv* or experience* or attitude* or percept* or perspect* or perciev* or view* or value* or belief*) adj3 treatment*).ti,ab.
27. ((improv* or experience* or attitude* or percept* or perspect* or perciev* or view* or value* or belief*) adj3 management*).ti,ab.
28. ((improv* or experience* or attitude* or percept* or perspect* or perciev* or view* or value* or belief*) adj3 healthcare*).ti,ab.
29. or/18-28
30. 10 and 17 and 29

Embase:

1. dementia/
2. Frontotemporal Dementia/
3. Dementia, Multi Infarct/
4. Dementia, Vascular/
5. Alzheimer Disease/
6. Lewy Body Disease/
7. dement*.ti.
8. alzheimer*.ti.
9. ((memory adj2 problem*) or (memory adj2 difficult*) or (memory adj2 issue*)).ti.
10. or/1-9
11. Qualitative Research/
12. Evaluation Studies/
13. exp Interview/
14. ((focus adj2 group*) or interview*).ti,ab.
15. (view* or perspective* or opinion* or attitude* or qualitative or ethnograph*).ti,ab.
16. or/11-15
17. Attitude to Health/
18. Professional-Family Relations/
19. Professional-Patient Relations/
20. exp Attitude of Health Personnel/
21. Holistic Care/
22. Doctor Patient Relation/
23. Nurse Patient Relationship/
24. ((improv* or experience* or attitude* or percept* or perspect* or perciev* or view* or value* or belief*) adj3 care).ti,ab.
25. ((improv* or experience* or attitude* or percept* or perspect* or perciev* or view* or value* or belief*) adj3 service*).ti,ab.
26. ((improv* or experience* or attitude* or percept* or perspect* or perciev* or view* or value* or belief*) adj3 practice*).ti,ab.
27. ((improv* or experience* or attitude* or percept* or perspect* or perciev* or view* or value* or belief*) adj3 treatment*).ti,ab.
28. ((improv* or experience* or attitude* or percept* or perspect* or perciev* or view* or value* or belief*) adj3 management*).ti,ab.
29. ((improv* or experience* or attitude* or percept* or perspect* or perciev* or view* or value* or belief*) adj3 healthcare*).ti,ab.
30. or/17-29
31. 10 and 16 and 30

PsychInfo:

1. dementia/
2. Frontotemporal Dementia/
3. Dementia, Multi Infarct/
4. Vascular Dementia/
5. Alzheimer Disease/
6. Lewy Body Disease/
7. dement*.ti.
8. alzheimer*.ti.
9. ((memory adj2 problem*) or (memory adj2 difficult*) or (memory adj2 issue*)).ti.
10. or/1-9
11. Qualitative Research/
12. Empirical Methods/
13. Group Discussion/
14. Interviews/
15. ((focus adj2 group*) or interview*).ti,ab.
16. (view* or perspective* or opinion* or attitude* or qualitative or ethnograph*).ti,ab.
17. or/11-16
18. ((improv* or experience* or attitude* or percept* or perspect* or perciev* or view* or value* or belief*) adj3 care).ti,ab.
19. ((improv* or experience* or attitude* or percept* or perspect* or perciev* or view* or value* or belief*) adj3 service*).ti,ab.
20. ((improv* or experience* or attitude* or percept* or perspect* or perciev* or view* or value* or belief*) adj3 practice*).ti,ab.
21. ((improv* or experience* or attitude* or percept* or perspect* or perciev* or view* or value* or belief*) adj3 treatment*).ti,ab.
22. ((improv* or experience* or attitude* or percept* or perspect* or perciev* or view* or value* or belief*) adj3 management*).ti,ab.
23. ((improv* or experience* or attitude* or percept* or perspect* or perciev* or view* or value* or belief*) adj3 healthcare*).ti,ab.
24. Health Personnel Attitudes/
25. Health Care Delivery/
26. Health Care Utilization/
27. Health Attitudes/
28. Client attitudes/
29. Client centered Therapy/
30. or/18-29
31. 10 and 17 and 30

CINAHL:

1. TI (dement* or alzheimer* or "memory N2 problem*" or "memory N2 issue*" or "memory N2 difficult*")
2. MH Dementia+
3. 1 or 2
4. AB (view* or perspective* or opinion* or attitude* or qualitative or ethnograph* or interview* or "focus group*")
5. MH Qualitative Studies OR MH Focus Groups OR MH Interviews+
6. 4 or 5
7. AB (((improv* or experience* or attitude* or percept* or perspect* or perciev* or view* or value* or belief*) N3 care))
8. AB (((improv* or experience* or attitude* or percept* or perspect* or perciev* or view* or value* or belief*) N3 service*))
9. AB (((improv* or experience* or attitude* or percept* or perspect* or perciev* or view* or value belief*) N3 practice*))
10. AB (((improv* or experience* or attitude* or percept* or perspect* or perciev* or view* or value* or belief*) N3 treatment*))
11. AB (((improv* or experience* or attitude* or percept* or perspect* or perciev* or view* or value* or belief*) N3 management*))
12. AB (((improv* or experience* or attitude* or percept* or perspect* or perciev* or view* or value* or belief*) N3 healthcare*))
13. MH Professional-Patient Relations+ OR MH Professional-Family Relations OR MH Attitude to Health OR MH Patient Centered Care OR MH Holistic Care OR MH Attitude of Health Personnel
14. 7 or 8 or 9 or 10 or 11 or 12 or 13
15. 3 and 6 and 14

C. Study quality ratings

Supplementary table of CASP quality ratings

Study	1	2	3	4	5	6	7	8	9	10	
1. Andersen et al. (2008)	Y	Y	Y	Y	Y	C	Y	Y	Y	Y	**9**
2. Bogardus et al. (1998)	Y	Y	Y	Y	Y	C	C	Y	Y	N	**7**
3. Bowes et al. (2003)	Y	Y	Y	Y	Y	C	Y	C	Y	Y	**8**
4. Bronner et al. (2016)	Y	Y	Y	Y	Y	C	Y	Y	Y	Y	**9**
5. Bunn et al. (2015)	Y	Y	Y	Y	Y	Y	Y	Y	Y	Y	**10**
6. Clarke et al. (2010)	Y	Y	Y	Y	Y	C	Y	Y	Y	Y	**9**
7. Di Gregorio et al. (2015)	Y	Y	Y	Y	Y	C	Y	Y	Y	Y	**9**
8. Dickins et al. (2018)	Y	Y	N	Y	Y	Y	Y	Y	Y	N	**8**
9. Foley et al. (2017)	Y	Y	Y	Y	Y	C	Y	Y	Y	Y	**9**
10. Forbes et al. (2011)	Y	Y	Y	Y	Y	C	Y	Y	Y	N	**8**
11. Forbes et al. (2013)	Y	Y	N	Y	Y	C	Y	Y	Y	N	**7**
12. Gilmour et al. (2003)	Y	Y	Y	Y	Y	Y	Y	Y	Y	Y	**10**
13. Groen van de Ven et al. (2017)	Y	Y	N	Y	Y	C	Y	Y	Y	Y	**8**
14. Groen van de Ven et al. (2018)	Y	Y	N	Y	Y	Y	Y	Y	Y	N	**8**
15. Low et al. (2013)	Y	Y	N	N	Y	C	Y	Y	Y	Y	**7**
16. Maidment et al. (2017)	Y	Y	Y	Y	Y	C	Y	Y	Y	Y	**8**
17. Martin et al. (2013)	Y	Y	N	Y	Y	C	C	Y	Y	Y	**7**
18. Newton et al. (2016)	Y	Y	N	Y	Y	C	Y	Y	Y	Y	**8**
19. Poppe et al. (2013)	Y	Y	N	Y	Y	C	Y	Y	Y	Y	**8**
20. Post et al. (2001)	Y	Y	Y	Y	Y	C	C	Y	Y	Y	**7**
21. Quinn et al. (2013)	Y	Y	Y	Y	Y	Y	Y	Y	Y	Y	**10**
22. Risco et al. (2015)	Y	Y	Y	Y	Y	Y	Y	Y	Y	Y	**9**
23. Rothera et al. (2008)	Y	Y	Y	Y	Y	C	Y	Y	Y	N	**8**
24. Spector et al. (2011)	Y	Y	N	Y	Y	C	Y	Y	Y	Y	**8**
25. St-Amant et al. (2012)	Y	Y	Y	Y	Y	C	Y	Y	Y	Y	**8**
26. Stephan et al. (2018)	Y	Y	Y	Y	Y	C	Y	Y	Y	Y	**9**
27. Tilburgs et al. (2018)	Y	Y	Y	Y	Y	C	Y	Y	Y	Y	**9**
28. Toot et al. (2013)	Y	Y	Y	Y	Y	C	C	Y	Y	Y	**8**
29. Ward-Griffin et al. (2012)	Y	Y	Y	Y	Y	C	C	Y	Y	Y	**8**

1. Was there a clear statement of the aims of the research?
2. Is a qualitative methodology appropriate?
3. Was the research design appropriate to address the aims of the research?
4. Was the recruitment strategy appropriate to the aims of the research?
5. Was the data collected in a way that addressed the research issue?
6. Has the relationship between researcher and participants been adequately considered?
7. Have ethical issues been taken into consideration?
8. Was the data analysis sufficiently rigorous?
9. Is there a clear statement of findings?
10. Is there consideration for the value of the research?
